# Self-Assembled Monolayers As a Tool to Investigate the Effect of Surface Chemistry on Protein Nucleation

**DOI:** 10.1021/acs.cgd.2c01377

**Published:** 2023-03-31

**Authors:** Fiora Artusio, José A. Gavira, Roberto Pisano

**Affiliations:** † Department of Applied Science and Technology, Politecnico di Torino, 24 Corso Duca degli Abruzzi, 10129 Torino, Italy; ‡ Laboratorio de Estudios Cristalográficos, Instituto Andaluz de Ciencias de la Tierra (Consejo Superior de Investigaciones Científicas-Universidad de Granada), Avenida de las Palmeras 4, 18100 Armilla, Granada, Spain

## Abstract

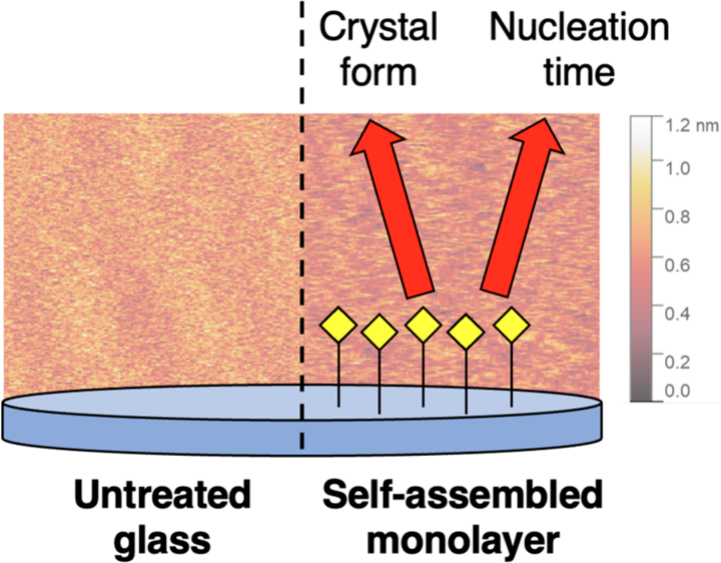

Modified surfaces like siliconized glass are commonly used to support protein crystallization and facilitate obtaining crystals. Over the years, various surfaces have been proposed to decrease the energetic penalty required for consistent protein clustering, but scarce attention has been paid to the underlying mechanisms of interactions. Here, we propose self-assembled monolayers that are surfaces exposing fine-tuned moieties with a very regular topography and subnanometer roughness, as a tool to unveil the interaction between proteins and functionalized surfaces. We studied the crystallization of three model proteins having progressively narrower metastable zones, i.e., lysozyme, catalase, and proteinase K, on monolayers exposing thiol, methacrylate, and glycidyloxy groups. Thanks to comparable surface wettability, the induction or the inhibition of nucleation was readily attributed to the surface chemistry. For example, thiol groups strongly induced the nucleation of lysozyme thanks to electrostatic pairing, whereas methacrylate and glycidyloxy groups had an effect comparable to unfunctionalized glass. Overall, the action of surfaces led to differences in nucleation kinetics, crystal habit, and even crystal form. This approach can support the fundamental understanding of the interaction between protein macromolecules and specific chemical groups, which is crucial for many technological applications in the pharmaceutical and food industry.

## Abstract 2

Self-assembled monolayers (SAMs) allow for precise control of surface chemistry and roughness. In this study, the crystallization of three model proteins, i.e., lysozyme, catalase, and proteinase K, was performed on SAMs to investigate heterogeneous nucleation. We show that the nucleation time, the number of crystals, and the crystal form can be modified depending on the terminal groups of SAMs.

## 1 Introduction

Vapor diffusion (VD) crystallization guarantees slow supersaturation rates thanks to the progressive removal of water from a drop containing protein and precipitants. The nucleation and growth of protein crystals can thus occur under mild conditions, favoring good-quality crystals’ harvesting. Over the years, complementary strategies for modifying the water evaporation rate have been proposed, including the use of capillaries,^1^ film barriers,^2^ and oil layers placed above the reservoir.^3^ Although little attention has been paid to the study of the effect of the surfaces supporting the drops, most experiments are conducted using siliconized coverslips. This superficial treatment leads to a well-rounded drop shape thanks to moderate surface hydrophobicity, thus allowing slow water removal. Few studies have investigated the effect of surfaces other than the siliconized glass on the crystallization of proteins.^4−8^ A chronological summary of proteins crystallized in the presence of heterogeneous substrates together with the proposed crystallization mechanism was reported by Fermani and co-workers^9^ in 2013, showing the diversity of possible interpretations of the results. Furthermore, surface patterning has been proven to be sufficient to induce nucleation.^10−13^

In our recent publication, we investigated the action of self-assembled monolayers (SAMs) immobilized on glass on the crystallization of a model active pharmaceutical ingredient (API), i.e., aspirin.^14^ SAMs turned out to have a dramatic impact on the nucleation step, both in terms of nucleation kinetics and preferential orientation of crystal faces nucleated on the surface. The use of uniform and flat surfaces as SAMs allows for the avoidance of surface heterogeneities in local charge or chemical composition, which can both affect nucleation.^9,15^ As far as biological macromolecules are concerned, the action of surfaces on nucleation is even more complex than for small molecules, since the effective alteration of the nucleation pathway of biomolecules by surfaces can only be achieved under specific conditions. In the metastable area of the phase diagram, only heterogeneous nucleation and crystal growth are allowed, thus excluding the overlap with homogeneous nucleation.

The interplay between different types of interactions, including electrostatics, hydrogen bonding, van der Waals, and hydrophobic interactions, determines the protein behavior in solution and the extent of interaction with external surfaces. In this scenario, the surface properties of the heteronucleant become of the utmost importance. Surface functionalization can often come along with altered surface roughness, which impedes a rigorous study of the effect of specific functional groups on protein dynamics. Surface topography plays a key role since roughness decreases the energetic penalty required by heterogeneous nucleation.^15,16^ Conversely, the energetic demand increases with the contact angle between the surface and the drop of protein plus precipitant solution.^17^ Studies on hydrophobic SAMs made of hydrocarbon chains of variable length and different terminal groups have shown that SAMs promoted faster crystallization rates and expanded the range of crystallization conditions compared to conventional coverslips. This ability was attributed to the inhibition of amorphous precipitation.^18,19^ The use of SAMs has also been reported to support the assembly of microscale crystals of redox proteins for constructing bioelectronic devices.^20^ Also, alternative approaches to foreign surfaces have been reported to promote crystallization. For example, the rational engineering of a protein surface to reduce the conformational entropy has been proposed.^21^

In the present paper, highly smooth SAMs have been used as supports for protein crystallization and to study the action of surface chemistry on nucleation phenomena avoiding surface roughness effects. Lysozyme, catalase, and proteinase K were crystallized on SAMs exposing various terminal groups to unveil the main mechanism governing surface–protein interaction.

## 2 Materials and Methods

### 2.1 Materials

All of the reagents employed for functionalizing coverslips made of borosilicate glass (D263M, Neuvitro, Vancouver, USA) were purchased from Sigma-Aldrich (Cesano Maderno, MI, Italy). 3-Mercaptopropyltrimethoxysilane (THIOL, 95%), 3-glycidyloxypropyltrimethoxysilane (GLY, ≥98%), 3-(trimethoxysilyl)propyl methacrylate (ACR, 98%), hydrogen peroxide (30 wt % in water, ACS reagent), sulfuric acid (ACS reagent, 95.0–98.9%), anhydrous toluene (<0.001% water, 99.8%), toluene (ACS reagent, ≥99.5%), and ethanol (puriss. p.a., absolute, ≥99.8%) were used for the synthesis of self-assembled monolayers. For the crystallization trials, lysozyme obtained from chicken egg white (62971, HEWL, three times crystallized powder, Sigma-Aldrich), catalase from bovine liver (C30, microcrystalline aqueous suspension, Sigma-Aldrich), and proteinase K (A3830, lyophilized, PanReac AppliChem, Barcelona, Spain) were selected. Before each crystallization test, protein concentration was measured via UV/vis spectrophotometry at 280 nm using 2.65, 1.48, and 1.42 mL·mg^–1^·cm^–1^ as extinction coefficients for HEWL, catalase, and proteinase K, respectively.

### 2.2 Synthesis of SAMs

The detailed synthesis optimization and characterization of SAMs is reported in our previous study.^22^ Briefly, all of the steps of the synthesis were carried out at room temperature. First, coverslips were sonicated twice in ethanol for 10 min and dried with nitrogen. Then, they were incubated in a 5:1 (H_2_SO_4_/H_2_O_2_) piranha solution for 1 h and thoroughly rinsed with deionized water. After drying, coverslips were immediately immersed in 0.054 M silane solutions in toluene for 15 h. To complete the synthesis, three washing steps with toluene, toluene/ethanol (1:1), and ethanol and blow-drying with nitrogen were performed to remove unreacted silanes and solvent residues. SAMs topography was measured by Atomic Force Microscopy (AFM, Solver NANO, NT-MDT Spectrum Instruments, Russia) in tapping mode. The scanned area was 1 μm^2^, at 256 lines per scan. SiN_4_ tips were used, and the cantilever frequency was 0.8 Hz. Gwyddion software (ver. 2.51, Czech Metrology Institute) was used to process the images after the analyses.

### 2.3 Crystallization of Proteins on Functionalized Surfaces

All of the protein solutions were filtered with 0.22 μm pore-size low protein-binding membrane filters. Initially, the crystallization conditions of three proteins, i.e., HEWL, catalase, and proteinase K, were investigated in the absence of SAMs inside mushroom crystallizers.^15^ Equal volumes of protein and precipitant solutions were mixed to form 6 μL drops. The reservoir was filled with 5 mL of precipitant solution. As reported in Table 1, 5−80 mg/mL HEWL were buffered in 50 mM Na acetate at pH 4.5 and mixed with variable amounts of stock NaCl solutions. Catalase was dissolved in 50 mM K phosphate at pH 7.0 to 1.8–3.7 mg/mL concentration and crystallized with 15% PEG4000. Finally, 10–30 mg/mL proteinase K in 50 mM HEPES at pH 7.0 were crystallized with either 0.25 M NaNO_3_ in 25 mM Na citrate or 1.7 M (NH_4_)_2_SO_4_. The crystallizer was sealed with vacuum grease, incubated at 20 °C, and periodically checked (five times per day, from 8 am to 8 pm, during the first 2 days of the experiment and twice per day on the following days) with an optical microscope (AZ100 Nikon, Germany) for the presence of crystals.

**Table 1:** Crystallization Conditions (Referred to Stock Solutions) and Protein Net Charge at Corresponding pH for HEWL, Catalase, and Proteinase K (the Ratio between Protein and Precipitant Stock Solutions Was 1)

protein	protein solution	precipitant solution	protein net charge, C
HEWL	5–80 mg/mL in 50 mM Na acetate pH 4.5	4–15 wt % NaCl	+13.0
Catalase	1.8–3.7 mg/mL in 50 mM K phosphate pH 7.0	15 wt % PEG4K	–11.9
Proteinase K	10–30 mg/mL in 50 mM HEPES pH 7.0	0.25 M NaNO_3_, 25 mM Na citrate	+1.7
		1.7 M (NH_4_)_2_SO_4_	

**Figure 1:**
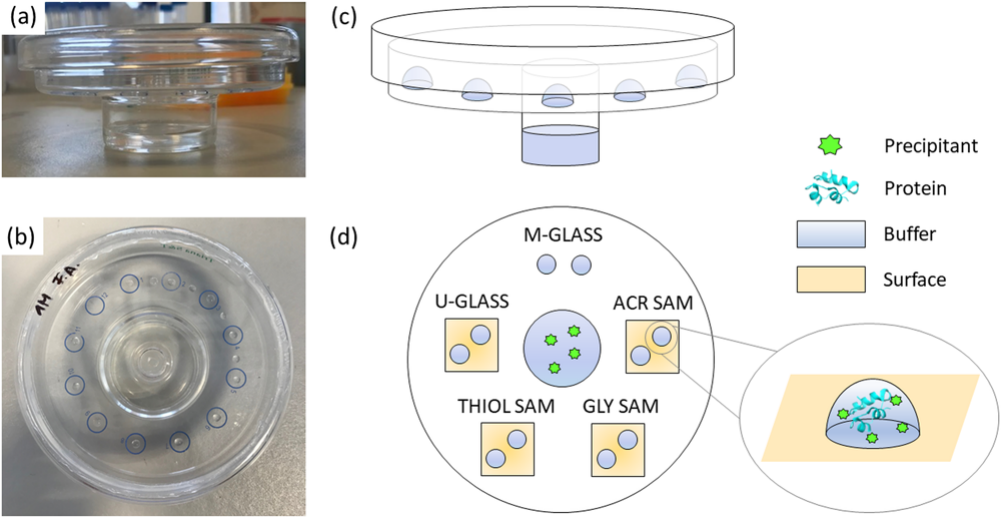
(a) Front view, (b) top view, and (c) schematics of the mushroom crystallizer used for VD crystallization. (d) Schematics of the experimental setup used to study protein crystallization on surfaces. Two drops containing protein and precipitant were carefully deposited on each surface (m-glass, u-glass, THIOL SAM, GLY SAM, and ACR SAM). An enlarged view of a drop is also represented.

Once the optimal conditions had been identified, protein crystallization was performed on functionalized surfaces. One untreated coverslip and three coverslips functionalized with different SAMs were carefully placed inside mushroom crystallizers according to the setup illustrated in Figure 1. Mushroom glass (m-glass) and untreated glass coverslips (u-glass) were selected as reference surfaces to compare the results obtained on SAMs. The experiments were carried out inside mushroom crystallizers to guarantee identical dynamics for all of the drops placed on different substrates (u-glass, m-glass, THIOL SAM, GLY SAM, ACR SAM). All of the surfaces were secured to the crystallizer surface with vacuum grease. Equal volumes of protein and precipitant solutions were mixed to form 6 μL drops that were carefully placed on the surfaces inside the mushroom crystallizer and incubated at 20 °C. Two drops were deposited on each surface. Experiments were performed in duplicate.

Catalase crystals were diffracted at the ID23-1 and ID30A-3 beamlines of the European Synchrotron Radiation Facility (ESRF), Grenoble, France. Crystals were fished out from the drops with the help of LithoLoop (Molecular Dimensions Inc., Apopka, FL, USA), immediately flash cooled in liquid nitrogen, and stored for data collection.

### 2.4 Crystallization of Proteins on Functionalized Surfaces at Different pH

Further tests inside mushroom crystallizers were carried out with HEWL and catalase varying the pH of the precipitant solution. Experiments were performed both without and with SAMs. In regard to stock solutions, the HEWL concentration was 60 mg/mL in 50 mM Na acetate at pH 4.5, the precipitation cocktail was made of 4 wt % NaCl in 400 mM Na acetate at a pH between 4.0 and 6.0. Drops were equilibrated against 4 wt % NaCl aqueous solution. The catalase concentration was set at 3.7 mg/mL. The precipitation cocktail was composed of 15 wt % PEG4000 in Milli-Q water for the reservoir and 15 wt % PEG4000 in 400 mM K phosphate at pH 6.0–8.0 for the precipitant solution of the drops. Experiments were performed in duplicate.

## 3 Results and Discussion

In the present study, self-assembled monolayers (SAMs) of silanes exposing thiol, methacrylate, and glycidyloxy terminations were grafted on glass. The chemical groups exposed by SAMs were selected so as to design an ideal platform to study nucleation. The three selected chemistries guaranteed highly reproducible and stable surface functionalization, comparable wettability, and subnanometer roughness, as determined by AFM (Figure S1). Alternative surface chemistries, such as amino-terminated SAMs, were excluded because of the instability of surface chemistry over time.^14,22^ Depending on the nature of the protein and its phase diagram, studying the action of surfaces on crystallization may be more or less challenging. Heterogeneous nucleation can only be studied when crystallization conditions fall in the metastable zone of the protein phase diagram. Therefore, the width of the metastable zone is related to the probability of observing heterogeneous nucleation events. In the present study, we selected three handleable model proteins, HEWL, proteinase K, and catalase, having different metastable zones, as qualitatively described in previous nucleation studies.^23^ HEWL has a wide metastable zone. Conversely, proteinase K has a very narrow metastable zone, thus being in principle insensitive to the presence of foreign surfaces. Catalase was selected as a protein showing an intermediate behavior.

### 3.1 HEWL

**Figure 2:**
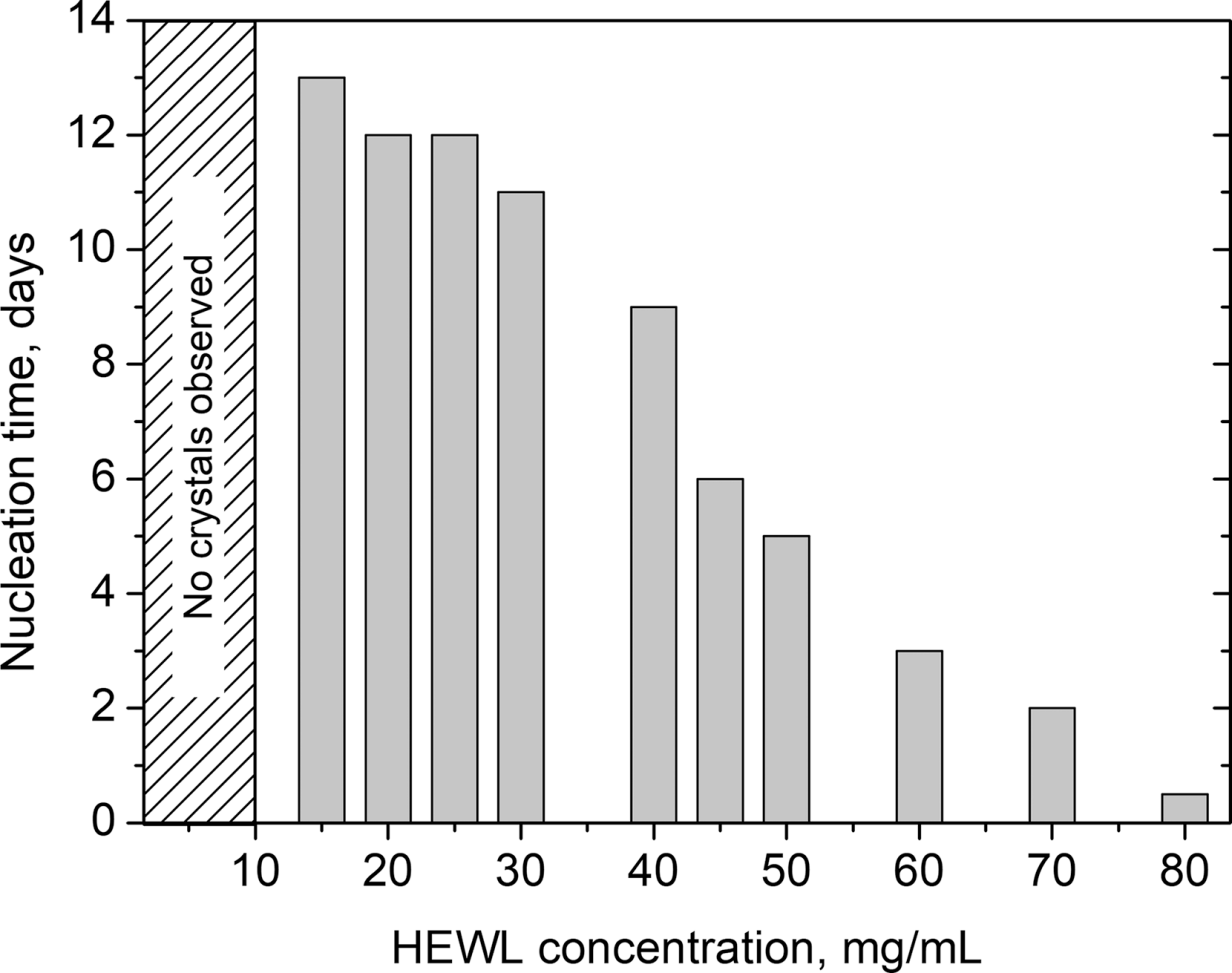
Nucleation time as a function of HEWL concentration in the mother protein solution.

The crystallization of HEWL on modified surfaces was first studied since such a protein represents a well-established model for nucleation studies and has a wide metastable zone. As a first step, screening over the nucleation time of HEWL on an m-glass surface was carried out inside mushroom crystallizers to identify the optimal nucleation kinetics and transpose such conditions to experiments involving modified surfaces. HEWL concentration was increased from 5 to 80 mg/mL in 50 mM Na acetate at pH 4.5 and crystallized with 4 wt % NaCl. The nucleation time was defined as the time elapsed to observe the first crystals in the drops by optical microscopy observation. As outlined in Figure 2, a fine-tuning of the time needed to detect the first crystals inside the drops was achieved. HEWL concentrations below 10 mg/mL did not lead to crystals within an experimental observation time of 20 days. Protein concentrations between 45 and 60 mg/mL led to crystals within 3–6 days and were selected for pursuing the study on SAMs. The nucleation time had to be slow enough to allow the system equilibration, the diffusion of protein macromolecules, and their interaction with the surface. At the same time, the heterogeneous nucleation area of the phase diagram had to be tackled. Higher protein concentrations led to too short nucleation times, potentially masking the effect of a foreign surface, and promoting bulk crystallization. In contrast, lower protein concentrations significantly delayed nucleation, increasing the probability of encountering perturbations due to vibrations, protein oxidation, contamination, etc., during the experiments on modified surfaces, thus potentially compromising the results. A parallel study was also performed on salt concentration: 60 mg/mL HEWL was mixed with 4 to 15 wt % NaCl (see Figure S2). As expected, the increase in the ionic strength shortened the nucleation time: few crystals were grown with 4 wt % NaCl after 3 days, whereas massive crystallization was obtained with 15 wt % NaCl after a few hours. It was believed that the extent of interaction between protein and salt under the latter conditions would prevail over the interaction between protein and foreign surfaces. Therefore, the optimal salt concentration for crystallization on SAMs was set at 4 wt %.

Having selected the proper protein concentration range, two crystallization experiments were performed on THIOL, GLY, and ACR SAMs setting the HEWL concentration at 45 and 57 mg/mL. Thanks to the relatively high protein concentration and low salt concentration, the crystallization conditions allowed for the presence of a significant amount of macromolecules available to interact with the surfaces. Two reference surfaces were selected to compare the results: EtOH-cleaned glass coverslips (u-glass) and the mushroom glass (m-glass). Such a need was due to the different contact angle displayed by the protein solution on the two types of glass: approximately 40° and 60° contact angles were obtained on u-glass and m-glass, respectively. Surface hydrophobicity affects the drop shape and, thus, the evaporation rate. From this perspective, all of the SAMs had a comparable contact angle to u-glass to rigorously compare the nucleation kinetics regardless of the differences in evaporation rates due to surface hydrophobicity.

**Figure 3:**
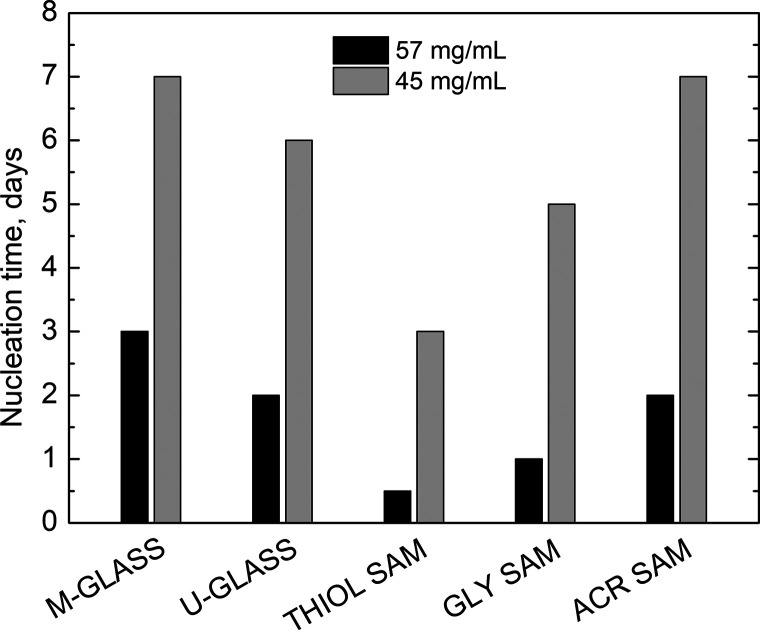
Comparison among the HEWL nucleation times on different surfaces at 57 (black bars) and 45 mg/mL (gray bars) HEWL and 4 wt % NaCl, considering stock solutions.

The nucleation time obtained on different SAMs and on the two reference glasses is reported in Figure 3 for the two HEWL concentrations studied. Considering the higher protein concentration (black bars), the shortest nucleation time was observed on THIOL SAMs (approximately 12 h), followed by GLY SAMs (1 day) and ACR SAMs (2 days). Experiments performed with lower protein concentration (gray bars) confirmed such a tendency, and in particular the THIOL SAM induction. At lower supersaturation, the gap between the nucleation times obtained on SAMs was enlarged, whereas it was not modified for the reference glasses (1 day difference). Therefore, the mere effect of glass hydrophobicity was flattened at low supersaturation, whereas the effect of surface chemistry was predominant.

The inducing ability of THIOL SAMs was attributed to the early aggregation of HEWL macromolecules thanks to the decrease in energetic penalty required for nucleation promoted by the surface. It has been previously reported that when aggregates are formed on a surface and the bond angles between protein molecules match those of a protein crystal, they can evolve into nuclei and grow.^24,25^ We hypothesized that the charge–charge interactions between THIOL SAMs (negatively charged, surface zeta potential, SZP = −42.3 mV^22^) and HEWL (positively charged, see Figure S3) could attract more protein molecules toward the surface and increase the local supersaturation. ACR and GLY SAMs, on the other hand, carried fewer negative charges on the surface (SZP = −21.3 mV and −24.7 mV, respectively) and had less impact on nucleation.

**Figure 4:**
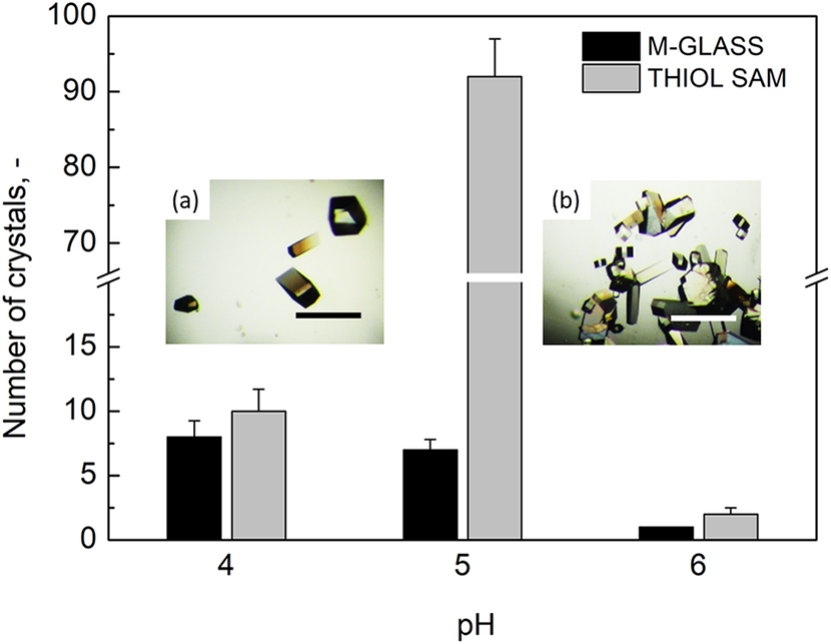
Average number of crystals nucleated in drops deposited on m-glass and THIOL SAMs as a function of pH. Embedded are two representative micrographs illustrating crystal density on (a) m-glass at pH 5 and on (b) THIOL SAMs at pH 5. The scale bar is 500 μm.

In order to get further insight in the hypothesized mechanism of nucleation induction of THIOL SAMs on HEWL crystallization, experiments were repeated varying the pH. In this sense, the pH of the protein buffer was varied from 4.0 to 6.0, while keeping constant the rest of the conditions. To test the suitability of such a system for HEWL crystallization, we preliminarily carried out an HDVD screening, which confirmed the appearance of well-faceted crystals at all pH values (see Figure S4). The HEWL nucleation time on m-glass and on THIOL SAMs as a function of pH is reported in Table 2. The nucleation-inducing ability of THIOL SAMs was preserved over the whole range of pH since the nucleation time of HEWL on THIOL SAMs was significantly shortened for all of the tested conditions. The trend of crystal density as a function of pH was in good agreement with that of the nucleation time, as shown in Figure 4. The marked action of THIOL SAMs on nucleation was underlined by the high number of crystals obtained at pH 5.0 (approximately 90 crystals). The nucleation-inducing ability of THIOL SAMs was preserved even at pH values that are not optimal for HEWL crystallization. For example, at pH 6.0 it was possible to observe crystals already after 1 day, in contrast to 13 days observed for the control. This result confirmed the promotion of early HEWL aggregation and the surface-stabilization of the critical nucleus promoted by THIOL SAMs.

**Table 2:** HEWL Nucleation Time on m-Glass and on THIOL SAMs As a Function of pH (The Observation Time Was 15 Days)

pH	nucleation time on m-glass	nucleation time on THIOL SAM	lysozyme net charge, C
4.0	3 days	12 h	+15.5
5.0	1 day	6 h	+10.8
6.0	13 days	1 day	+8.9

### 3.2 Catalase

Catalase was the second protein tackled in this study. Catalase crystallization is more challenging since the protein can easily undergo oxidation and lose its activity, resulting in a more difficult process to control. As for HEWL, a preliminary screening on nucleation time was performed. However, a precise tuning over nucleation time was not achieved as, after 1 day, crystals were observed in all of the drops prepared starting from 1.8 to 3.7 mg/mL catalase and 15 wt % PEG4000. Further reduction in protein concentration hindered the crystal formation, rather than tuning nucleation time, and it would be detrimental to ensure protein/surface interaction.

**Figure 5:**
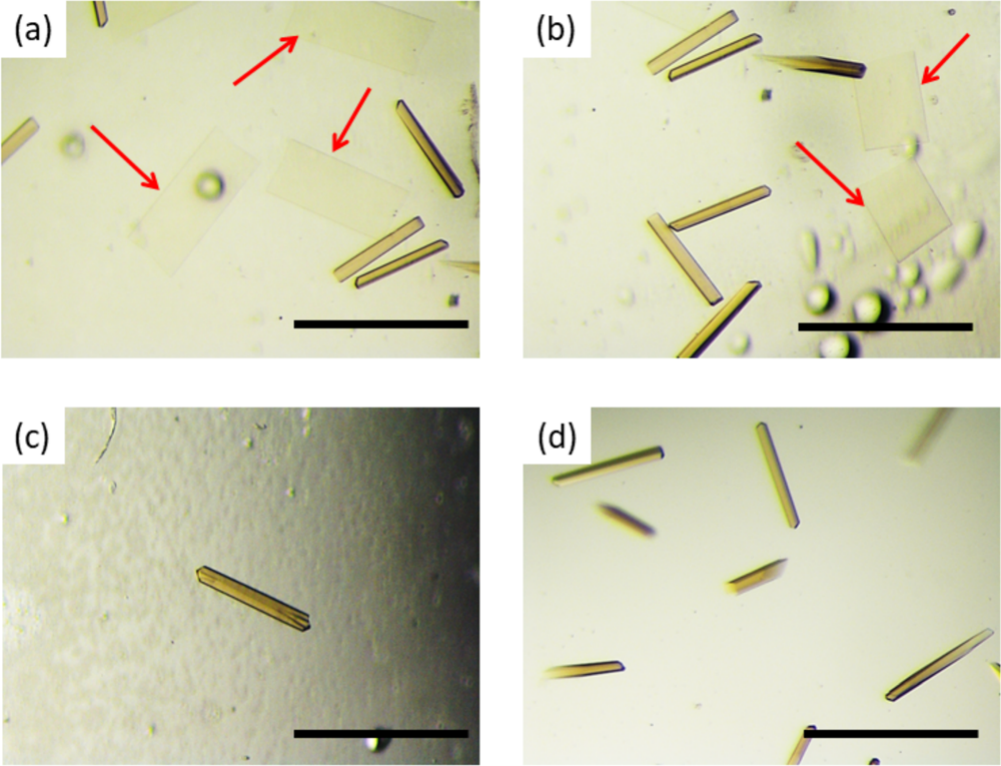
(a, b) Catalase crystals grown on GLY SAMs. Two nucleation events were observed and led to bar and plate-like (indicated by the arrows) crystals. Reference catalase crystals grown on (c) m-glass and (d) coverslips. Scale bar is 500 μm.

Low supersaturation conditions were tested on the surfaces to maximize the impact of SAM chemistries (2.4 mg/mL catalase, 15 wt % PEG4000). After 12 h, crystals were obtained on all of the surfaces, except on THIOL SAMs (observation time = 10 days). The average nucleation time was by far shorter than for HEWL and, when nucleation occurred, it did in a comparable time window on all the surfaces. However, the morphology of the crystals was different, pointing to a clear effect of the SAMs on catalase crystallization. Elongated bar-like crystals (300–400 μm long) were generally obtained on m-glass, GLY, and ACR SAMs. A second population of thin plate-like crystals was observed only on GLY and ACR SAMs (highlighted by the arrows in Figure 5). These pieces of evidence pointed out that, in the absence of specific surface functionalization, catalase crystallized in the bulk to produce bar-like crystals and did not significantly interact with the surface. Conversely, when GLY or ACR SAMs were introduced into the system, the nucleation pathway of catalase was modified, and the growth of a second orthorhombic form was promoted. The plates were thin, whereas the two other dimensions were similar to the length of the bar-like crystals pointing out to a concomitant nucleation. The coexistence of these two polymorphs somehow modified the system, allowing the nucleation and growth of yet another crystal form belonging to the trigonal space group (Table 3). This crystal form had only two monomers in the asymmetric unit compared to the four monomers of both orthorhombic forms. Trigonal crystals were smaller than the orthorhombic ones and appeared after a while, indicating that this crystal form is not kinetically favored under these conditions. Although we cannot attribute this new polymorph to the direct effect of the surface, we indirectly observed it thanks to the whole dynamics of the system imposed by the SAMs.

**Table 3:**
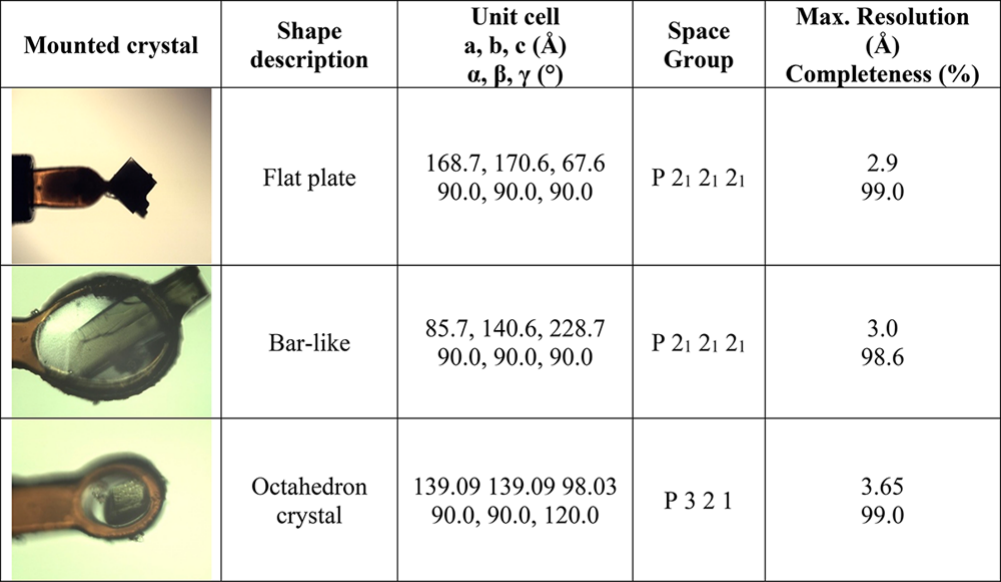
Catalase Crystals Nucleated on SAMs and Diffracted at Synchrotron Beamline^a^

a The measured unit cells, space groups, maximum resolution, and completeness are reported. Loop size is 0.2, 0.2, and 0.06 mm for the flat-plate, bar, and octahedron crystals, respectively.

When higher supersaturation conditions were employed (3.7 mg/mL catalase, 20 wt % PEG4000), massive crystallization was obtained on all of the surfaces (Figure S5). Under these conditions, bulk nucleation prevailed, and only bar-like crystals were obtained, even on THIOL SAMs. Catalase was also crystallized varying the pH between 6.0 and 8.0. Results, reported in Table S1, show that fast crystallization (within 12 h) occurred both on m-glass and THIOL SAMs for pH above 7.0, whereas longer nucleation times were required to obtain crystals at pH 6.0. At this pH, crystals appeared first on THIOL SAMs (3 days) and then on m-glass (5 days). Thus, the inhibitory effect observed with THIOL SAMs at low supersaturation was overturned in an inducing effect at high supersaturation. This apparently contradictory result was explained considering that, at pH 6.0, catalase net charge is close to 0 coulomb (see Figure S3). In the absence of charge–charge repulsion, which is dominant at higher pH, nucleation could be induced by the nonspecific interactions.

Overall, even if appreciable differences in nucleation kinetics were not observed, it was possible to collect information on the early stages of catalase crystallization on different surfaces by analyzing the morphology of the crystal populations.

### 3.3 Proteinase K

The crystallization of proteinase K was last investigated. Preliminary HDVD screening tests were performed with sodium nitrate or ammonium sulfate to look for the most appropriate crystallization conditions to analyze SAMs effects. However, as shown in Table S2 and Figure S7, high supersaturation led to quasi-immediate protein crystallization with high nucleation density and therefore small crystal size. Moderate supersaturation, on the other hand, slowed down nucleation time to 12 h, whereas slightly lower supersaturations (proteinase K = 15 or 10 mg/mL) pushed the system within the metastable zone with much higher waiting time (i.e., no crystals after 72h). Because of the narrow metastability zone of proteinase K, bulk nucleation always prevails, masking the effect of surfaces, as already observed in the case of agarose.^23^

## 4 Conclusion

In the present study, the action of surface chemistry on the nucleation of three model proteins, i.e., lysozyme, catalase, and proteinase K, was investigated. SAMs displaying thiol, methacrylate, and glycidyloxy groups were used because of their extremely high surface smoothness coupled with controlled chemistry. These surface features mitigated the potential influence of surface roughness decoupling the chemical and physical influence of the surface on nucleation. The interaction between the exposed chemical groups and the protein macromolecules was exploited to tune the nucleation step. We demonstrated that surfaces cannot be generally applied to any kind of protein since the interaction is specific for each protein and depends on the protein itself, the charge (pH), and the level of supersaturation. The action of surfaces was clearly discerned for lysozyme. With a wide metastable zone, this protein was an ideal model for studying specific interactions with SAMs. THIOL SAMs strongly promoted nucleation thanks to electrostatic matching, as confirmed by crystallization experiments over a wide pH range. When the direct tackling of nucleation was not feasible, as in the case of catalase, we analyzed the crystal population’s morphology to correlate the surface’s action to nucleation. Such a technique may support the discovery of new polymorphs thanks to the early stabilization of prenucleation clusters by a selective interaction with the surface and help the fundamental understanding of the interaction between proteins and specific chemical groups. The change of the dynamic of the drop allowed us to get a third polymorph in the case of catalase extending the benefits of using SAMs. Lastly, when the metastability zone was even narrower, as for proteinase K, the action of SAMs was completely leveled off.

## Supporting Information Available

Additional details on AFM topographies of SAMs; the proteins’ net charge vs pH; the crystallization of lysozyme, catalase, and proteinase K (PDF)

## Abbreviations

ACR: 3-(trimethoxysilyl)propylmethacrylate
API: active pharmaceutical ingredient
EtOH: ethanol
GLY: 3-glycidyloxypropyltrimethoxysilane
HD: hanging drop
HEWL: hen egg white lysozyme
PEG: polyethylene glycol
SAM: self-assembled monolayer
SZP: surface zeta potential
THIOL: 3-mercaptopropyltrimethoxysilane
UV: ultraviolet
VD: vapor diffusion
VIS: visible
