# Supplementary material for: Self-Assembled Monolayers As a Tool to Investigate the Effect of Surface Chemistry on Protein Nucleation
Source: Cryst Growth Des. 2023 Mar 31;23(5):3195–201. doi: 10.1021/acs.cgd.2c01377 (PMC10162442; doi:10.1021/acs.cgd.2c01377)
Supplement: Supplementary file 1 — cg2c01377_si_001.pdf [file cg2c01377_si_001.pdf]

ELECTRONIC SUPPORTING INFORMATION

Self-assembled monolayers as a tool to investigate the effect of surface chemistry on protein nucleation

Fiora Artusio[1], José A. Gavira[2], Roberto Pisano[1]*

[1] Department of Applied Science and Technology, Politecnico di Torino, 24 corso Duca degli Abruzzi, 10129 Torino, Italy

[2] Laboratorio de Estudios Cristalográficos, Instituto Andaluz de Ciencias de la Tierra (Consejo Superior de Investigaciones Científicas-Universidad de Granada), Avenida de las Palmeras 4, 18100 Armilla, Granada, Spain

*Correspondence to: fiora.artusio@polito.it

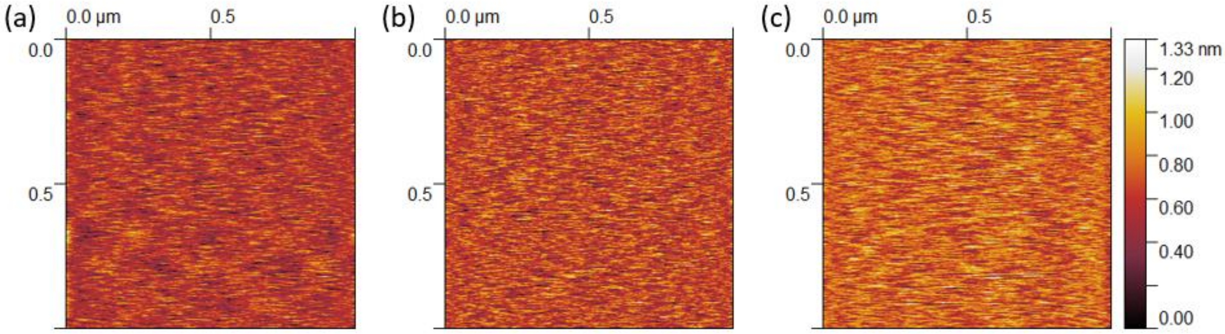

Figure S1. AFM topographies of (a) THIOL, (b) GLY, and (c) ACR SAMs.

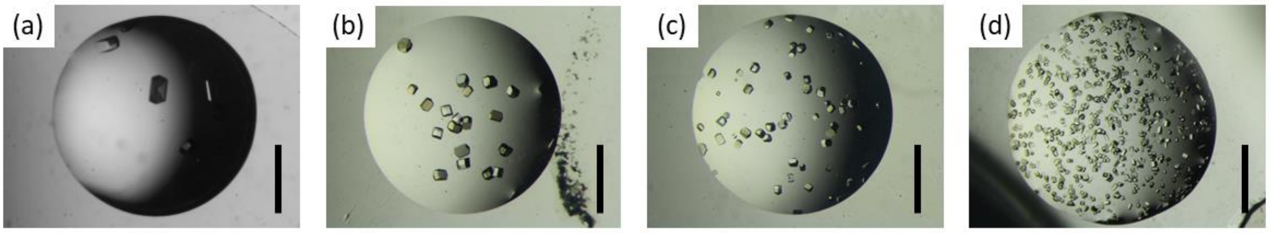

Figure S2. HEWL crystals grown in drops deposited on m-glass at progressively higher supersaturation. Protein concentration was 60 mg/mL and NaCl was (a) 4, (b) 7, (c) 11, and (d) 15wt% considering stock solutions. The scale bar is 1 mm.

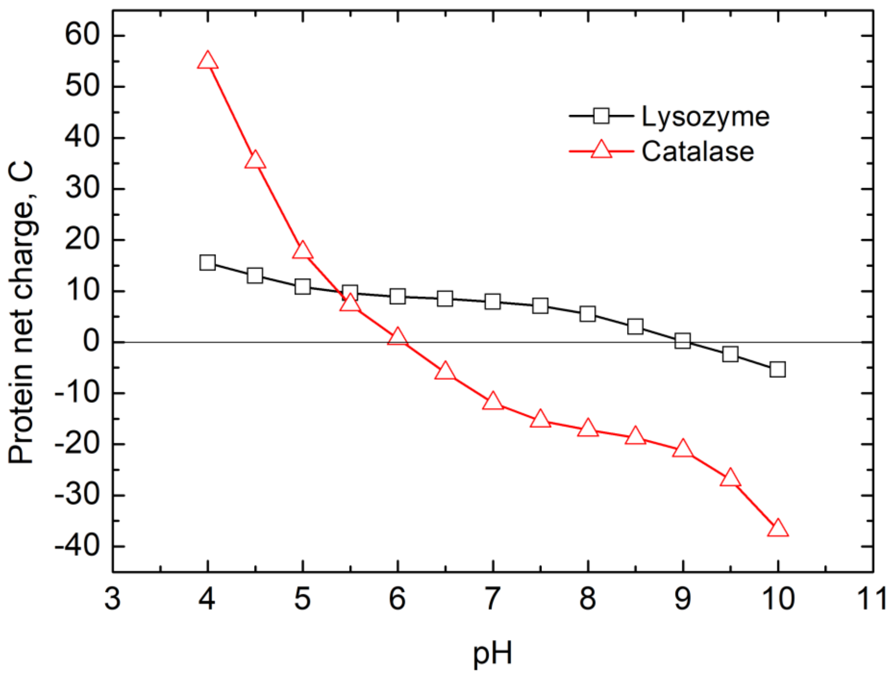

Figure S3. The protein net charge as a function of pH for lysozyme and catalase. Calculations were made with Protein Calculator (v3.4), knowing the sequence of amino acids for each protein.

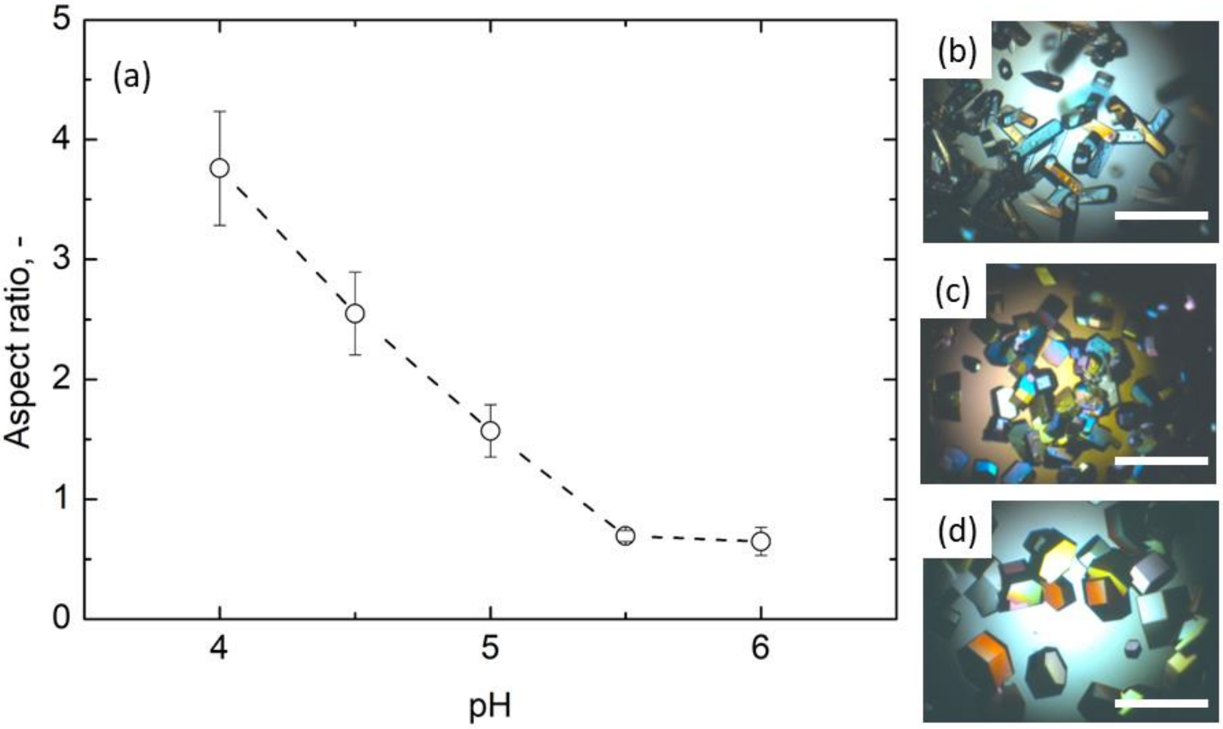

Figure S4. (a) The aspect ratio of HEWL crystals vs. pH. Error bars refer to standard deviation. HEWL concentration was 50 mg/mL in 50 mM Na acetate pH 4.5, the precipitation cocktail was made of 4.5wt% NaCl buffered in 400 mM Na acetate at defined pH. The ratio between protein and precipitant solutions was 1. The total volume drop was 3 μL. On the right side, representative micrographs of drops referring to pH (b) 4, (c) 5, and (d) 6 are illustrated. The scale bar is 500 μm.

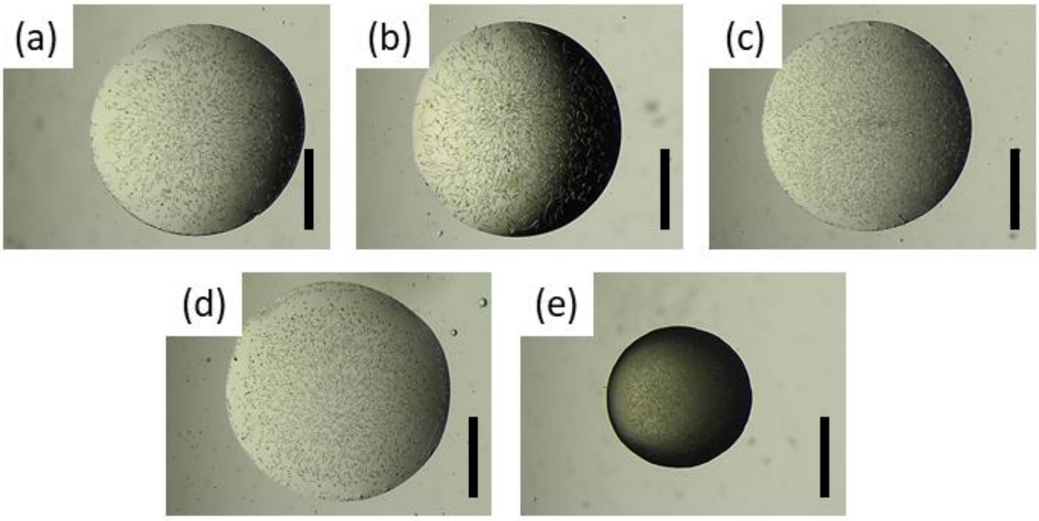

Figure S5. Massive crystallization of catalase carried out at high supersaturation (3.7 mg/mL of catalase, 20wt% PEG4000) on (a) THIOL, (b) ACR, (c) GLY SAMs, (d) u-glass, and (e) m-glass. The scale bar is 1 mm.

Table S1. The nucleation time of catalase on m-glass and THIOL SAMs, as observed at different pH (3.7 mg/mL of catalase, 20wt% PEG4000). The total volume drop was 6 μL.

**pH**	**Nucleation time on m-glass**	**Nucleation time on THIOL SAM**
6.0	5 days	3 days
6.5	1 day	12 hours
7.0	12 hours	12 hours
7.5	12 hours	12 hours
8.0	12 hours	12 hours

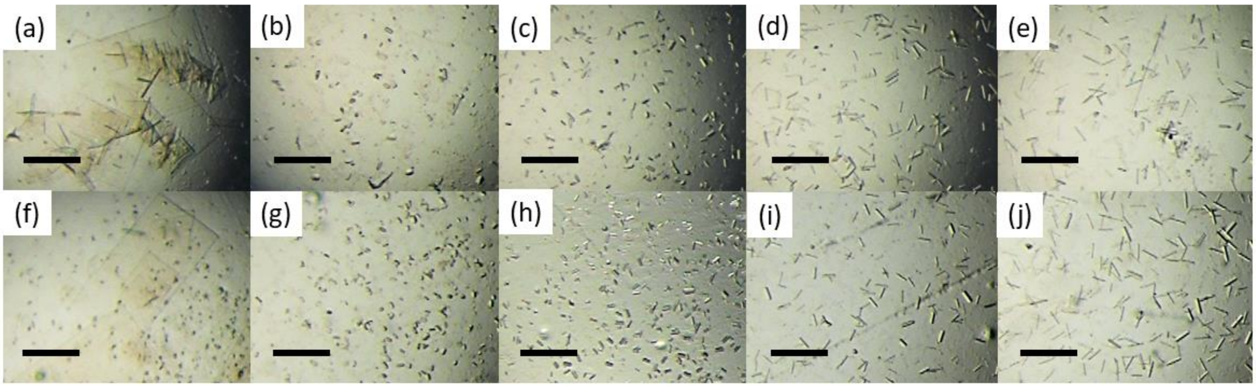

Figure S6. Representative catalase crystals grown at increasing pH. The protein stock concentration was 3.7 mg/mL. The precipitant solution was 20wt% PEG4000. The first row refers to drops deposited on m-glass and pH equal to (a) 6.0, (b) 6.5, (c) 7.0, (d) 7.5, and (e) 8.0. The second row refers to drops deposited on THIOL SAMs and pH equal to (f) 6.0, (g) 6.5, (h) 7.0, (i) 7.5, and (j) 8.0. The scale bar is 500 μm.

Table S2. The nucleation time for the various crystallization conditions screened for proteinase K. The total volume of the drops was 6 μL. The observation time was 15 days.

**Proteinase K, mg/mL**	**Precipitant**	**Nucleation time**
30	0.25 M $NaNO_3$<br>25 mM Na citrate	5 min
20	0.25 M $NaNO_3$<br>25 mM Na citrate	12 h
15	0.25 M $NaNO_3$<br>25 mM Na citrate	-
10	0.25 M $NaNO_3$<br>25 mM Na citrate	-
30	1.7 M $(NH_4)_2SO_4$	5 min
20	1.7 M $(NH_4)_2SO_4$	12 h
15	1.7 M $(NH_4)_2SO_4$	-
10	1.7 M $(NH_4)_2SO_4$	-

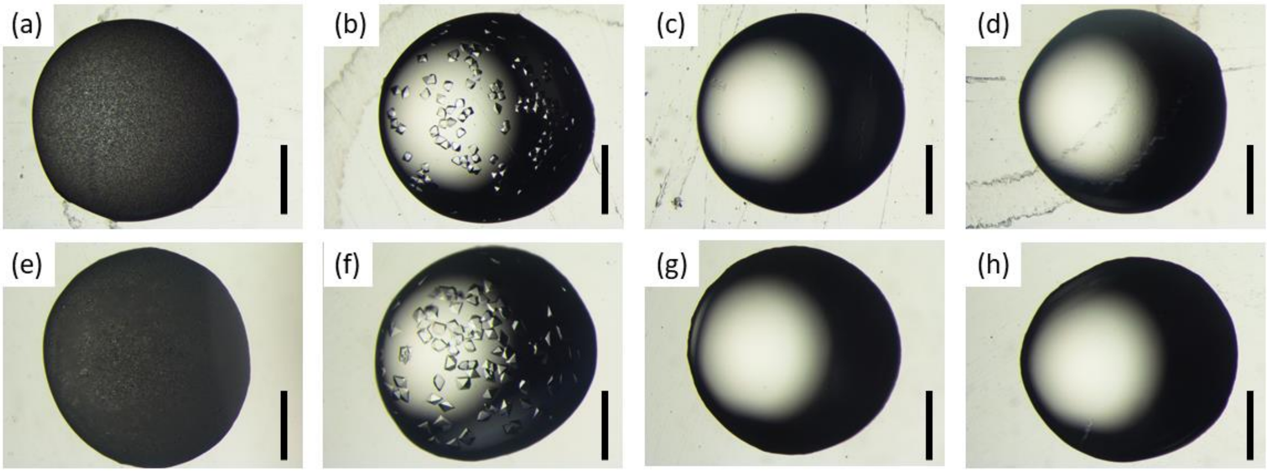

Figure S7. Outcome of crystallization of proteinase K (30, 20, 15, and 10 mg/mL) for experiments involving (a)-(d) 0.25 M $NaNO_3$ in 25 mM Na citrate and (e)-(h) 1.7 M $(NH_4)_2SO_4$. The scale bar is 1 mm.
